# A Preliminary Investigation into the Impact of a Pesticide Combination on Human Neuronal and Glial Cell Lines *In Vitro*

**DOI:** 10.1371/journal.pone.0042768

**Published:** 2012-08-03

**Authors:** Michael D. Coleman, John D. O'Neil, Elizabeth K. Woehrling, Oscar Bate Akide Ndunge, Eric J. Hill, Andre Menache, Claude J. Reiss

**Affiliations:** 1 School of Life and Health Sciences, Aston University, Birmingham, United Kingdom; 2 Antidote Europe, Perpignan, France; National Institutes of Health, United States of America

## Abstract

Many pesticides are used increasingly in combinations during crop protection and their stability ensures the presence of such combinations in foodstuffs. The effects of three fungicides, pyrimethanil, cyprodinil and fludioxonil, were investigated together and separately on U251 and SH-SY5Y cells, which can be representative of human CNS glial and neuronal cells respectively. Over 48h, all three agents showed significant reductions in cellular ATP, at concentrations that were more than tenfold lower than those which significantly impaired cellular viability. The effects on energy metabolism were reflected in their marked toxic effects on mitochondrial membrane potential. In addition, evidence of oxidative stress was seen in terms of a fall in cellular thiols coupled with increases in the expression of enzymes associated with reactive species formation, such as GSH peroxidase and superoxide dismutase. The glial cell line showed significant responsiveness to the toxin challenge in terms of changes in antioxidant gene expression, although the neuronal SH-SY5Y line exhibited greater vulnerability to toxicity, which was reflected in significant increases in caspase-3 expression, which is indicative of the initiation of apoptosis. Cyprodinil was the most toxic agent individually, although oxidative stress-related enzyme gene expression increases appeared to demonstrate some degree of synergy in the presence of the combination of agents. This report suggests that the impact of some pesticides, both individually and in combinations, merits further study in terms of their impact on human cellular health.

## Introduction

Pesticide use is integral in world agriculture. In the Californian wine industry, over twenty thousand tonnes of pesticide are used per annum, including more than 150 different agents [1]. Although pesticides can improve yields and restrict dietary threats to human health such as mycotoxins [2], there remains a long-standing concern over the impact of the sheer quantity of pesticide usage on the environment [3], [4]. There also appears to be some association between human toxicity and the weight of pesticide application, as commercially farmed areas in the wine industry increase in size [1].

Amongst the various pesticides deployed, fungicides are particularly important in the wine grape industry and their usage in various guises has been documented since Roman times [5], [6]. The application of combinations of different pesticides and especially those with novel modes of action has been largely driven by widespread fungal resistance; this has resulted from the pressure of historical biocidal usage, as well as increased efficacy against pests and the promotion of better yields [7], [8]. In the wine industry, the persistence of biocides, leading to detectable contamination of the finished product, is highly variable and depends on many factors [9]–[11]. The process of wine production generally reduces pesticide contamination in comparison with that of grapes, although this depends on the physiochemical properties of the agents used [9] and it is now apparent that wines sourced from Europe contain multiple residues of many different pesticide agents in measurable quantities [12], [13]. Indeed, some combinations of agents such as fludioxonil and cyprodinil can be resistant to destruction during wine processing [14]. There are concerns that these levels of contamination might exceed published European Union maximum residue levels (MRL; [9], [15]), although these limits are not strictly enforced [16].

Considerable research has been carried out on the impact of pesticides on various non-target flora and fauna [17], [18] and there is evidence that many biocidal agents lack specificity. Indeed, they can exert significant mammalian toxicity that may be synergistic where combinations of different agents are deployed [19]–[21]. There is also evidence that pesticide exposure can increase the risk of genotoxicity and this may result in specific malignancies [22], [23]. In addition, pesticide exposure can be neurotoxic [24] and is even considered a risk factor in the development of neurodegenerative conditions such as Parkinsonism [25], [26].

However, in view of the use of many combinations of biocides, their known persistence through foodstuff processing and their potential neurological human impact, there has been little investigation into the possible synergistic biological effects on human neuronal and glial cellular systems. In the present study, we have utilized two cell lines, gliomal U251 and neuroblastomal SH-SY5Y cells, which model the basic cell types of the human central nervous system, the astrocytes and neurones. In this preliminary study, we have explored the effects of a combination of three commonly used biocides (pyrimethanil, ciprodinil and fludioxonil) on cell viability, mitochondrial health and generation of oxidative stress, alone and in combination.

## Methods

### Materials and Reagents

Tissue culture flasks, pipettes and 96-well microtitre plates were obtained from Corning Costar (Lowell, MA, USA). All types of cell culture media, as well as foetal bovine serum (FBS) and L-glutamine were purchased from Invitrogen (Paisley, UK). Penicillin and streptomycin, as well as the biocidal agents cyprodinil, fludioxonil and pyrimethanil were supplied by Sigma-Aldrich (Poole, Dorset, UK). The CellTiter Blue™ viability assay was purchased from Promega (Southampton, UK).

### Cell Culture

SH-SY5Y (human neuroblastoma) cells (ECACC no. 94030304; [27] were maintained in Roswell Park Memorial Institute 1640 containing Glutamax I® (Invitrogen), supplemented with 10% (v/v) FBS, 1% non-essential amino acids (NEAA) and 1% (v/v) penicillin/streptomycin (100 units penicillin- G/ml and 10 mg/ml streptomycin). U251-MG cells [28] were maintained in and Hams F10 medium with 25 mM HEPES, supplemented with 10% heat-inactivated FBS, 2 mM l-glutamine and 1% (v/v) PenStrep solution. The cells were passaged using 0.25% Trypsin, 0.1 mM EDTA when they had reached approximately 80–90% of confluency.

### Study Design

The pesticides were dissolved completely in stock DMSO solutions and serially diluted in media prior to addition to the cells. The agents were studied singly in the various assays and in combination across a concentration range of 0–1 mM. in 100 µl phenol red free DMEM (containing 2 mM L-Glutamine, 10% v/v FBS, 1% (v/v) PenStrep solution, in triplicate wells, for 48 hours at 37°C, 5% CO_2_. Cells were incubated with increasingly cytotoxic concentrations of pesticides with the aim of the calculation of IC_50_ values for the various assays. In the combination studies, all three agents were added so that the same net weight of pesticide was present in the incubations as those containing single agents. The vehicle controls contained DMSO alone.

### Cell Viability Assay in the presence of Pesticides

Pyrimethanil, ciprodinil and fludioxonil effects on cell general viability were evaluated initially through the CellTiter Blue™ (resazurin assay; Promega) viability assay singly and in combinations. Prior to toxin treatment, SHSY5Y and U251 cells were plated in 96-well microtitre plates at concentrations of 4 or 5×10^5^ cells/ml respectively, in a 100 µl volume. After incubation overnight, the medium was removed and the cells were maintained in the presence of 100 µl medium supplemented with increasing concentrations of pesticides. After a 48-hour incubation period, the cell culture medium was completely replaced with phenol red free DMEM to which 1% v/v CellTiter-Blue™ (Promega) solution was added. Cultures were incubated for 4 hours at 37°C/5% CO_2_ and then read at 620 nm. Untreated negative controls were run together with the treated cells. Plates with reagent only served as background controls. Reciprocal absorbance values were calculated and the percent viability was expressed as the value in the presence of toxicant as a percentage of that in the medium only control (which was set as 100%).

### JC-1 Mitochondrial membrane potential assay

The mitochondrial membrane potential (MMP) decrease may be determined using JC-1 dye which only enters cells possessing healthy mitochondria with polarised membranes, where it forms aggregates which exhibit a fluorescent shift from green to red. The JC-1 assay was performed according to Gartlon *et al.* [29]. Briefly, following a 48 hour exposure to the test chemicals, the cell culture medium in each well was completely replaced with 100 µl phenol red free DMEM containing 5 µM JC-1 (Sigma, UK), prepared from a 1 mM JC-1 stock in DMSO. Following incubation of the plates for 30 min at 37°C, 5% CO_2_, the medium was removed, the cells washed three times with PBS and 100 µl PBS was added to each well. The red substrate fluorescence intensity was then read at 544 nm/590 nm (excitation/emission). The results were expressed as a percentage of the fluorescence value from the medium only control well (which was set as 100%).

### Total glutathione assay

The total glutathione assay was performed according to as described by Schuglia *et al.*, [30]. Briefly, following a 48 hour exposure to increasingly cytotoxic concentrations of test chemical, the cell culture medium in each well was completely replaced with 25 µl/well of a 5% (w/v) sulfosalicylic acid (SSA) solution and the plates subjected to three freeze-thawing steps. A working solution of 0.2 mM NADPH and 0.52 mM DTNB was prepared in sodium dihydrogen phosphate assay buffer (0.1 M sodium dihydrogen phosphate, pH 7.5 with 0.15 mM EDTA) and a 5 U/ml GSR enzyme solution was prepared by dilution of glutathione reductase from baker's yeast (500 U in 3.6 M ammonium sulphate; Sigma, UK) with assay buffer. Aliquots of 10 µl from each SSA treated well were removed in triplicate to a new 96-well plate and 165 µl of NADPH/DTNB working solution was added to each well, followed by incubation at 37°C for 15 min. GSR enzyme solution at 40 µl was added, the plate incubated at room temperature for 5 min whilst protected from light and the A_405_ was then measured. The total glutathione content of samples was determined from standard curves generated with known amounts of GSH using the same procedure and expressed as a percentage of the medium only control well (which was set as 100%).

### ATP Assay

ATP levels were determined using the ATP luminescence assay kit from Invitrogen (Paisley, UK), as per the manufacturer's protocol. Briefly, following a 48 hour exposure increasingly cytotoxic concentrations of test chemical, a 10 µl aliquot from each well was transferred to a black 96-well plate and 90 µl of an ATP standard solution (containing 1 mM dithiothreitol, 0.5 mM D-luciferin, and 2.5 µl of 5 mg/ml firefly luciferase stock solution per 10 ml of the 1X reaction buffer provided) added. The plate was then incubated for 15 min at 37°C, 5% CO_2_ and then the luminescence was measured. The ATP content of samples was determined from standard curves generated with known amounts of ATP using the same procedure and expressed as a percentage of the medium only control well (which was set as 100%).

### Data analysis

If applicable, GraphPad Prism was used to define the concentration that gave a 50% reduction in assay response (IC_50_ value) and standard error of the mean (SEM) for each pesticide and the combination. All experiments were conducted on three separate occasions in quadruplicate and one-way ANOVA followed by the relevant post-hoc test was used to assess for differences between IC_50_ values, with significance accepted at the 5% level (p<0.05). All figures and tables are presented as mean ± SEM.

### Real-Time PCR

Total RNA was extracted using Tri® Reagent, quantified by spectrophotometry and treated with DNase and an RNase inhibitor (Promega, Southampton, UK). 1 µg of total RNA was reverse transcribed using AMV reverse transcriptase (Promega, Southampton UK) and oligo dT_15_ primers (Promega, Southampton, UK). Real-Time PCR: cDNAs were amplified in a standard 40-cycle SYBR® green real-time PCR reaction using optimised sequence specific primers for caspase-3, superoxide dismutase (SOD) and glutathione peroxidase (GSHPx) supplied by PrimerDesign Ltd (Southampton UK), according to the manufacturer's instructions.

GAPDH was selected as a stably expressed gene for normalisation of test gene expression after GeNorm analysis (PrimerDesign Ltd, Southampton UK). The comparative CT method was used to calculate the relative quantification of gene expression. The following formula was used to calculate the relative amount of the transcripts in the chemical treated samples (treat) and the vehicle-treated samples (control), both of which were normalized to the endogenous controls. ΔΔCT = ΔCT (treat) – ΔCT (control) for biological RNA samples or ΔΔCT = ΔCT (HBRR) – ΔCT (UHRR) for reference RNA samples. ΔCT is the difference in CT between the target gene and endogenous controls by subtracting the average CT of controls from each replicate. The fold change for each treated sample (relative to the control sample (or UHRR) = 2^−ΔΔCT^. Fold changes in gene expression using the comparative CT method and statistical analysis were determined using the freely available Relative Expression Software Tool (REST 2009, www.qiagen.com).

## Results and Discussion

### Impact of the agents alone and in combination on cell viability

**Figure 1:**
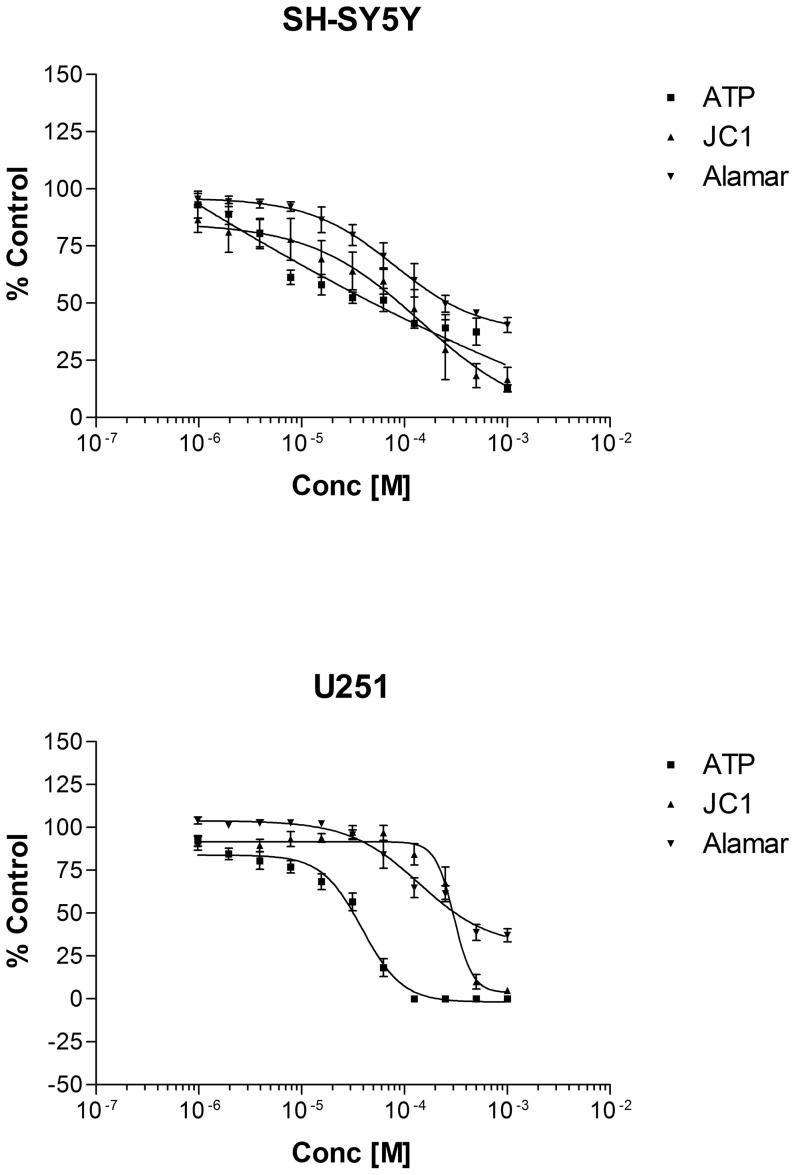
CellTiter Blue™ viability, ATP and JC-1 determinations expressed as a percentage of control values after the incubation of SH-SY5Y and U251 cells with a mixture of pyrimethanil, cyprodinil and fludioxonil over 1–1000 µM over 48 h (n = 3 per determination).

These studies are summarized on Table 1. In terms of the CellTiter Blue™ results, both cell lines exhibited similar levels of sensitivity with regard to the combination of pesticides, as well as to fludioxonil and cyprodinil alone, although pyrimethanil was significantly more toxic in the U-251 line compared with the SH-SY5Y cells (P<0.001). For both cell lines, the combination was the most toxic, followed by cyprodinil and either fludioxonil (SH-SY5Y) or pyrimethanil (U-251; P<0.001). There was a marked significant difference between the CellTiter Blue™ assay IC_50_'s and those of the JC-1 assay in the SH-SY5Y cells (P<0.001), but not in the U251 cells with the exception of the cyprodinil measurements. With the JC-1 assay, in both cell lines, cyprodinil and the combination were the most toxic (P<0.001). In order of descending toxicity, fludioxonil in the SH-SY5Y cells and pyrimethanil on the U251 cells were the next most potent, followed by pyrimethanil in the neuronal and fludioxonil in the glial line were the least toxic (P<0.001). All the pesticides showed an extremely potent reduction in ATP levels, which was approximately ten-fold greater than their effects in the CellTiter Blue™ viability assay (P<0.001). However, ATP reductions were not significantly different with regard to cell line or individual agent, with the minor exception of cyprodinil values, which were significantly lower (P<0.05) than those of pyrimethanil in the SH-SY5Y cells. From Figure 1, it is apparent that the pattern of toxicity of the pesticide combination, with respect to the mitochondrial effects (JC-1 and ATP assays) differed markedly between the neuronal and glial cell lines. Whilst the viability, ATP and JC-1 data assumed an approximately linear increase in toxicity (Figure 1), the U251 cells appeared to demonstrate a more sigmoidal appearance in terms of the ATP and JC-1 data, with the glial lines exhibiting apparently greater resistance to mitochondrial membrane attrition compared with the SH-SY5Y cells (P<0.001).

**Table 1:** Respective IC_50_ values **(µM)** after exposure of U251 and SH-SY5Y cell lines to pyrimethanil, cyprodinil and fludioxonil singly and in combination using three viability assays (CellTiter Blue™, JC-1 and ATP).

Assay	Pesticide	U251 (µM IC_50_)	±SEM	SH-SY5Y (µM IC_50_)	±SEM
**CellTiter Blue**	Pyrimethanil	598.1^a^ *** ^, ^ c *** &	22.5	822.3^a^ *** ^, ^ b *** ^,^ c *** &	14.2
	Cyprodinil	332.3^b^ ** ^,^ c ***	28.8	304.4^b^ *** ^,^ c ***	28.2
	Fluodioxonil	801.5^a^ *** ^, ^ c ***	46.7	708.1^a^ *** ^, ^ b *** ^, ^ c ***	42.6
	**Combination**	**257.2**	26.8	**269.9** b ***	26.2
**JC-1**	Pyrimethanil	526.3^a^ ***	47.6	144.3^a^ ***	9.6
	Cyprodinil	203.7	18.5	40.5	1.7
	Fluodioxonil	877.2^a^ ***	13.5	100.6^a^ ***	23.6
	**Combination**	**273.6**	20.3	**63.9**	13.9
**ATP**	Pyrimethanil	31.8	2.2	44.5@	6.3
	Cyprodinil	28	3.4	33@	2.2
	Fluodioxonil	32.7	1.3	34.2	6.1
	**Combination**	**30.9**	2.8	**39.3**	2.7

* P<0.05,

** P<0.001,

*** P<0.001.

a) denotes significant difference between single pesticide and combination IC50 values for each of the 3 assays.

b) denotes significant difference between respective CellTiter Blue™ and JC-1 IC50 values.

c) denotes significant difference between respective CellTiter Blue™ and ATP IC50 values.

@ denotes P<0.05.

& denotes P<0.001.

**Figure 2:**
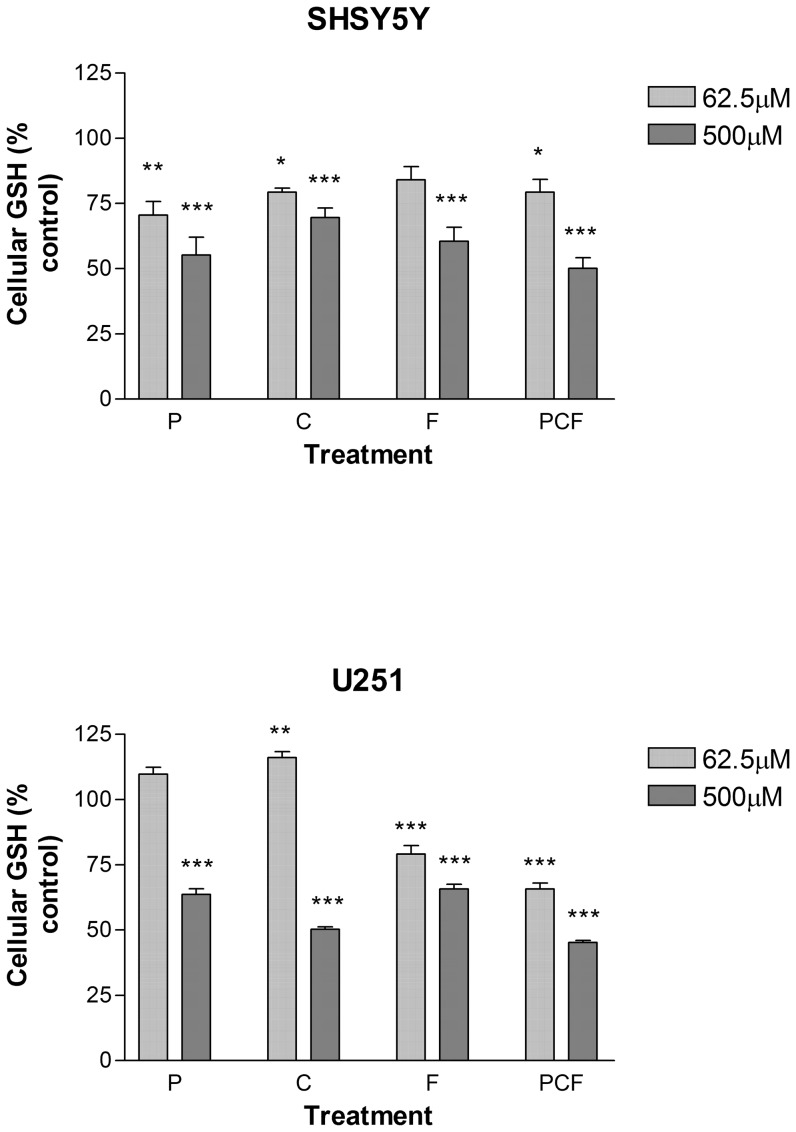
GSH determinations expressed as a percentage of control values after the incubation of SH-SY5Y and U251 cells with pyrimethanil, cyprodinil and fludioxonil used singly and in combination, over 1–1000 µM over 48 h (n = 3 per determination; *, P<0.05; **, P<0.01; ***, P<0.001).

Studies on GSH cellular content (Figure 2) indicated that the presence of the individual pesticides caused significant reductions in both cell lines at low agent concentrations; (cyprodinil and pyrimethanil in the SH-SY5Y cells and cyprodinil and fludioxonil in the U251 cells; P<0.05; 0.01). However, the pesticide combination showed significant reductions in GSH at low and high agent concentrations in both cell lines (P<0.001). Interestingly, cyprodinil at 62.5 µM appeared to show a net increase in GSH levels with respect to control (P<0.01). Overall, with the U251 cells even at the highest concentrations tested, GSH levels did not actually fall to 50% so an IC_50_ could not be calculated. With these cells, IC_50_ values for individual determinations for pyrimethanil, cyprodinil and fludioxonil ranged from 63.7–59.1%, although for the combination, an IC_50_ was determined to be 653+/−57.3 µM. In contrast, in terms of thiol depletion, the SH-SY5Y cells were more vulnerable than the glial cell line. IC_50_'s for thiol depletion for pyrimethanil and fludioxonil were not significantly different (770.1+/−66.5; and 849.9+/−49.9 µM), however, the values for combination (476.7+/−34.6 µM) as well as cyprodinil alone (535.8+/−54.8 µM) were not significantly different from each other, but they were significantly lower than the values for pyrimethanil and fludioxonil (P<0.001).

### qPCR gene changes

**Figure 3:**
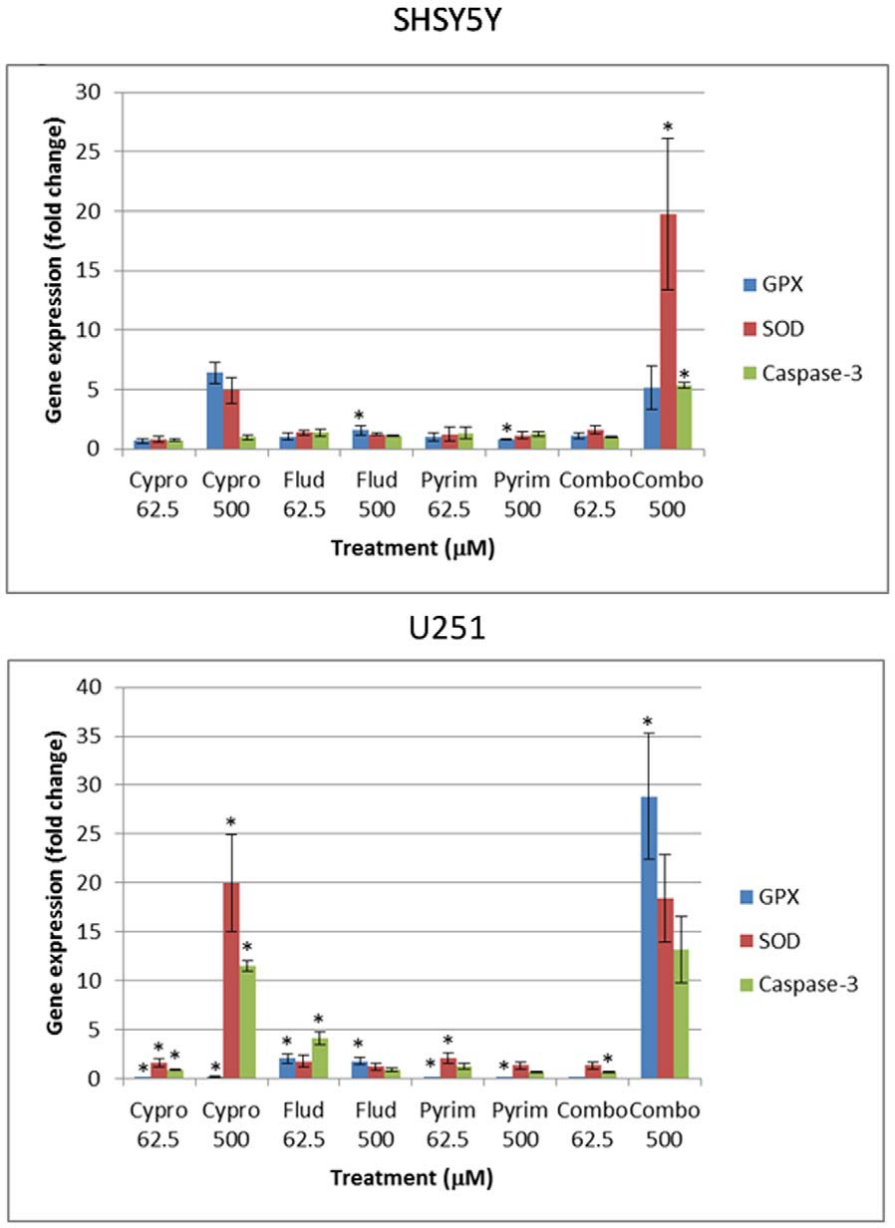
Fold changes in the expression of GSH peroxidase, superoxide dismutase and caspase-3 after the incubation of SH-SY5Y and U251 cells with pyrimethanil, cyprodinil and fludioxonil singly and in a mixture at a low (62.5 µM) and high (500 µM) exposure concentration over 48 h (n = 3 per determination; *, P<0.05).

Three enzymes that are strongly associated with oxidative stress, namely caspase-3, superoxide dismutase (SOD) and glutathione peroxidase (GSHPx), were studied using qRT-PCR. From Figure 3 it is clear that there was a marked difference between the two cell lines investigated, with the U251 cells demonstrating the most significant responses to the toxicants. With the U251 cells, cyprodinil and pyrimethanil treatment at both concentrations resulted in a marked decrease in the expression of GSHPx (P<0.05), whilst treatment with both concentrations of fludioxonil resulted in enhanced gene expression (P<0.05). In contrast, SOD expression was elevated to varying degrees by both concentrations of the individual compounds, with the most marked increase being 19.93-fold with the higher concentration of cyprodinil (P<0.05). Caspase-3 expression was enhanced by 11.53-fold and 4.1-fold in the presence of 500 µm cyprodinil and 62.5 µm fludioxonil, respectively (P<0.05). Whereas, treatment with the higher dose of pyrimethanil resulted in a marked decrease in expression (0.62 relative to control). In contrast to the varying increases and decrease in gene expression found with the compounds tested individually, the high dose of the compounds in combination resulted in a robust increase in the expression of GSHPx, SOD and caspase-3 by 28.54, 18.46 and 13.21-fold, respectively.

With the SH-SY5Y cells, fludioxonil and pyrimethanil showed only modest significant increases in expression at the higher concentration studied (P<0.05), although a 19.79 fold increase was recorded in SOD expression in the neuronal line in the presence of the combination (P<0.05). In the neuronal cells, a significant 5.3-fold increase in caspase-3 expression was also recorded (P<0.05). GSHPx expression was also found to be elevated in the presence of the higher concentration of the combination (5.12-fold), although this increase in gene expression was not found to be statistically significant.

### Pesticide effects on energy metabolism

The most striking aspect of the effects of cyprodinil, pyrimethanil and fludioxonil in this study, was that their toxicity towards the cell lines appeared to be directly linked with their potent impact on ATP production; this was in turn connected with their detrimental effects on the mitochondrial membrane potential, as indicated by the JC-1 assay. ATP production has a critical effect on cell viability and whether cell death is either necrotic or apoptotic and it is strongly dependent on the level of cellular ATP depletion [31]. In the human CNS, ATP depletion is a known route of neurotoxicity linked with several agents [32], [33]. In addition, all three pesticides are sufficiently lipophilic (log P's range from 2.8–4) to penetrate the human blood brain barrier and reach the CNS.

Negative effects on energy metabolism may also damage cell defence against reactive species. In this regard, the pesticides impacted cellular reducing power capability, but significantly, they also influenced the expression of several key protective enzymes. Such effects may be either direct, in terms of suppression or induction of a specific gene's expression, or they may be a cellular response to a secondary effect on cellular metabolism or health, such as oxidative stress and/or reduction in ATP levels. Although it is not always possible to distinguish between the two possible effects, in this report with the U251 cell line, cyprodinil and pyrimethanil directly impaired the expression of a major antioxidant defence enzyme (GSHPx) at low and high concentrations, whilst negatively affecting mitochondrial performance, an effect that should in itself lead to an increase in GSHPx expression. This impact on GSHPx occurred at pesticide concentrations that did not significantly impact thiol levels, or in the case of cyprodinil, when they were actually increased. It is conceivable that such an increase may have been linked with the absence of thiol demand that resulted from the fall in GSHPx expression. Although the pesticides caused some cellular response to increases in reactive species to occur, such as the increase in SOD expression, in both cell lines, the suppression of GSHPx expression in this report suggests that in the U251 line, cell defence could potentially be partially undermined by the pesticides. This effect was not apparent in the neuronal cells, where fludioxonil and pyrimethanil alone were associated with increases in GSHPx expression.

### Pesticide effects on Caspase-3 and detoxification response

Regarding caspase-3 activation, which is essential for the execution of apoptosis [34], cyprodinil and fludioxonil alone caused this effect, although the combination of pesticides with the U251 line showed very significant increases in the three key protective enzyme systems, GSHPx, SOD and caspase-3. Whilst this suggests that the combination of these agents was responsible for sufficient oxidative stress generation to elicit appropriate cellular defence gene responses in the U-251 cells, such a response to the combination was not evident in the SH-SY5Y line. At higher pesticide concentrations, it was apparent that thiol levels were also eroded in both cell lines and it is likely that this was a consequence of the combination of oxidative stress, attenuation of the ATP supply and mitochondrial toxicity. Although high consumption of thiols usually triggers a ‘thermostatic’ increase in thiol synthesis [35], this requires both reducing power and ATP to recycle GSH through GSSG reductase and *de novo* synthesis [36]. The impact of the pesticides on energy metabolism and antioxidant protection may have amplified the toxic effects of the pesticides alone and in combination.

### Context of findings in terms of modes of action and application

The effects of the pesticides seen in this report do not immediately appear to be linked with their mode of action in fungal control. The anilinopyrimidine pyrimethanil is a dual action fungicide, as it inhibits methionine synthesis and also perturbs cell wall degradation enzymes, thus retarding fungal invasion [37], [38]. Interestingly, although significantly more toxic than pyrimethanil in our hands, cyprodinil is actually a closely related structural anilinopyrimidine analogue. Indeed, cyprodinil is often mixed with fludioxonil, in such products as ‘Switch®’ which are used extensively in the wine industry [39]. Fludioxonil is a phenylpyrrole derivative of the antibiotic pyrrolnitrin [6] and is a potent phenylpyrrole protein kinase inhibitor; it can also interfere with signal transmission of osmoregulatory control in fungi [40]. Interestingly, the three antifungals are not perceived as especially toxic to mammals; oral LD_50_'s for these agents in rodents exceed 2–5 g/kg [41], [42] and their toxicity when used individually towards many benign species is often relatively low [43]. Overall, the toxicity of the agents singly and in combination seen in this report may be linked with commonality in basic cellular function between mammalian and other target species and this may have significantly eroded these agents' fungal specificity. Indeed, it is known that concerning mitochondrial structure and function, there are many similarities between mammalian and fungal systems [44]. In addition, a recent report has shown both cyprodinil and fludioxonil to inhibit human erythrocytic SOD, an effect that may also promote oxidative stress and cellular attrition [45].

### Pesticide cell-specific effects

The responses of the two cell lines explored in this study to the toxic impact of the pesticides differed markedly. The U251 line possesses considerable biotransformational activity [46] and is widely used as a cytotoxic model for a range of disparate agents such as ethanol and trimethyl tin chloride at concentrations which were within the range shown in human and other mammalian cellular systems [47]–[50]. The U251 line is also a relevant model of human astrocytes, demonstrating comparable susceptibility to glial toxins such as 6-hydroxydopamine in other models [47], [51]. The antioxidant responses in terms of protective enzyme expression and relatively higher resistance to thiol depletion compared with the SH-SY5Y cells, to some extent reflects the role of the astrocyte *in vivo*. Neurones are acutely vulnerable to damage caused by reactive oxygen and nitrogen-derived species and astrocytes play a key role in the oxidative defence of neurones and the preservation of their functionality [52].

The SH-SY5Y neuroblastoma line is used to model dopaminergic human neurones in several disease contexts [53], [54], as well as in the exploration of toxic cellular mechanisms and neurotoxin screening [55], [56]. The SH-SY5Y line has been reported as more sensitive to neurotoxins in comparison with hepatic and breast cancer lines [57]. However, these neurone-like cells are less robust than other transformed lines [57] and this was to some extent reflected in their greater degree of susceptibility to the pesticides in this report compared with the glioma cells.

### Findings in context of pesticide usage

The persistence of some of these agents is highly variable and is related to their immediate environment. With cyprodinil, this can range for a few days in vegetable crops [58], to several weeks along with fludioxinil in grape juice stored at 4°C [39]. Refrigeration of produce significantly extends their half-lives and can lead to breach of EU maximum residue limits [59]. Cyprodinil is also resistant to destruction in the vinification process [14] and only charcoal treatment will eliminate these agents from wine [60]. This leads to significant concentrations of these agents in wines and foodstuffs. In a study in Italian ‘ready meals’, there was an average of 2.4, but up to ten pesticides in every meal. Whilst this contamination, which included pyrimethanil and cyprodinil, did not exceed acceptable daily intakes (ADI) in adults, evidence was found that contamination was higher in children and teenagers and in some cases in these latter groups, the ADI's were actually exceeded [61]. 

Although pyrimethanil concentrations in some wines can range from about 0.6 to just over one micromolar [15], [62], this is well short of the levels required in the present study to cause mitochondrial toxicity in the two human cell lines. However, pyrimethanil and other related pesticides are found in some water supplies [63] as well as in many other foodstuffs. Therefore, in view of this report, further studies are necessary to determine the impact of individual pesticides and combinations of these agents on human cellular energy metabolism and oxidative stress production, particularly with regard to their potential ability to reach the human CNS.

## References

[1] Li W, Qin ZH, Zhang MH, Browde J (2011) An index method to evaluate growers' pesticide use for identifying on-farm innovations and effective alternative pest management strategies: a case study of winegrape in Madera County, California. J Zhejiang Uni-SCIENCE B (Biomedicine & Biotechnology) 12: 226–246. 10.1631/jzus.B0900264 PMC3048938 21370508

[2] LoCurto R, Pellicano T, Vilasi F, Munafo P, Dugo G (2004) Ochratoxin A occurrence in experimental wines in relationship with different pesticide treatments on grapes. Food Chem 84: 71–75.

[3] Thrupp LA (1990) Entrapment and escape from fruitless insecticide use: lessons from the banana sector of Costa Rica. Int J Environ Stud 36: 173–189.

[4] Reus J, Leendertse P, Bockstaller C, Fomsgaard I, Gutsche V, et al (2002) Comparison and evaluation of eight pesticide environmental risk indicators developed in Europe and recommendations for future use. Agric Ecosyst Environ 90: 177–187.

[5] González-Rodríguez RM, Cancho-Grande B, Gándara JS (2009) Multiresidue determination of 11 new fungicides in grapes and wines by liquid–liquid extraction/clean-up and programmable temperature vaporization injection with analyte protectants/gas chromatography/iontrap mass spectrometry. J Chromatogr A 1216: 6033–6042. 19576591 10.1016/j.chroma.2009.06.046

[6] Rosslenbroich HJ, Stuebler D (2008) *Botrytis cinerea* history of chemical control and novel fungicides for its management. J Plant Dis & Prot 115: 208–213.

[7] Engelmeier D, Hadacek F (2006) Antifungal natural products: assays and applications. Rai and Carpinella (eds.) in Naturally Occurring Bioactive Compounds. Elsevier B.V.

[8] Wing KD, Sacher M, Kagaya Y, Tsurubuchi Y, Mulderig L, et al (2000) Bioactivation and mode of action of the oxadiazine indoxacarb in insects. Crop Protect 19: 537–545.

[9] Likas DT, Tsiropoulos NG (2011) Fate of three insect growth regulators (IGR) insecticides (flufenoxuron, lufenuron and tebufenozide) in grapes following field application and through the wine-making process. Food Add Contam 28: 189–197. 10.1080/19440049.2010.542184 21318916

[10] Anli E, Vural N, Vural H, Gucer Y (2007) Application of solid-phase micro-extraction (SPME) for determining residues of chlorpyrifos and chlorpyrifos-methyl in wine with gas chromatography (GC). J Inst Brew 113: 213–218.

[11] Tsiropoulos NG, Miliadis GE, Likas DT, Liapis K (2005) Residues of spiroxamine in grapes following field application and their fate from vine to wine. J Ag Food Chem 53: 10091–10096. 10.1021/jf052162q 16366700

[12] Oliva J, Payá P, Cámara MA, Barba AJ (2007) Removal of famoxadone, fluquinconazole and trifloxystrobin residues in red wines: Effects of clarification and filtration processes. Environ Sci Health Part B-Pesticides Food Contaminants & Agricultural Wastes 42: 775–781. 10.1080/03601230701550964 17763033

[13] Jiménez JJ, Bernal JL, de Nozal MJ, Bernal J, Toribio L (2007) Persistance and degradation of metalaxyl, lindane fenvalarate and deltamethrin during the wine making process. Food Chem 104: 216–223.

[14] Cuš F, Cesnik HB, Bolta SV, Gergorcic A (2010) Pesticide residues in grapes and during vinification process. Food Control 21: 1512–1518.

[15] Anonymous, Pesticide Action Network Website, Available: http://pan-europe.info/Resources/Briefings/Message_in_a_Bottle.pdf Accessed 2012 July 13.

[16] European Food Safety Authority (2009) Reasoned Opinion of EFSA prepared by the Pesticides Unit on MRLs of concern for the active substance procymidone (revised risk assessment). EFSA Scientific Report 227: 1–26 Available: http://www.efsa.europa.eu/en/scdocs/doc/227r.pdf. Accessed 2012 July 13.

[17] Ensminger M, Bergin R, Spurlock F, Goh KS (2008) Pesticide concentrations in water and sediment and associated invertebrate toxicity in Del Puerto and Orestimba Creeks, California, 2007–2008. Env Mon Assess 4: 573–587. 10.1007/s10661-010-1552-y 20563640

[18] Caceres TP, Megharaj M, Naidu R (2011) Toxicity and transformation of insecticide fenamiphos to the earthworm, *Eisenia fetida* . Ecotoxicology 20: 20–28. 20882337 10.1007/s10646-010-0552-6

[19] Sidiropoulou E, Sachana M, Flaskos J, Harris W, Hargreaves AJ, et al (2011) Fipronil interferes with the differentiation of mouse N2a neuroblastoma cells. Toxicol Lett 201: 86–91. 21167920 10.1016/j.toxlet.2010.12.009

[20] Zhao Q, Ren QA, Chen JA, Qiu ZQ, Cao J (2011) Reproductive Toxicity in Male Mice Caused by Organic Extracts in Tap Water from the Jialing River in Ciongqing. China Birth Def. Res. Part B; Dev. Reproduct. Toxicol 92: 1–9. 10.1002/bdrb.20277 21312319

[21] Tuzmen MN, Candan N, Kaya E (2007) The evaluation of altered antioxidative defense mechanism and acetylcholinesterase activity in rat brain exposed to chlorpyrifos, deltamethrin, and their combination. Toxicol Mech Methods 17: 535–40. 20020880 10.1080/15376510701380463

[22] Alavanja MCR, Samanic C, Dosemeci M, Lubin J, Tarone R, et al (2003) Use of agricultural pesticides and prostate cancer risk in the Agricultural Health Study Cohort. Am J Epidemiol 157: 800–814. 12727674 10.1093/aje/kwg040

[23] Band PR Abanto Z, Bert J, Lang B, Fang R (2011) Prostate Cancer Risk and Exposure to Pesticides in British Columbia Farmers. The Prostate 71: 168–183. 20799287 10.1002/pros.21232

[24] Kimura H, Tsukagoshi NA, Yamaguchi T, Iijima A, Aoyama Y (2010) Relationships between cellular events and signaling pathways in various pesticide-affected neural cells. Toxin Rev 29: 43–50.

[25] Franco R, Li S, Rodriguez-Rocha R, Burn M, Panayiotidis MI (2010) Molecular mechanisms of pesticide-induced neurotoxicity: Relevance to Parkinson's disease. Chemico-Biological Interactions 188: 289–300. 20542017 10.1016/j.cbi.2010.06.003 PMC2942983

[26] Tanner CM, Kamel F, Ross GW, Hoppin JA, Goldman SM (2011) Rotenone, Paraquat and Parkinson's Disease. Environ Health Perspect 119: 866–872. 21269927 10.1289/ehp.1002839 PMC3114824

[27] Ross RA, Spengler BA, Biedler JL (1983) Coordinate morphological and biochemical interconversion of human neuroblastoma cells. J Natl Cancer Inst 71: 741–747. 6137586

[28] Wikstrand CJ, Bigner DD (1981) Hyperimmunization of non-human primates with bcg-cw and cultured human glioma-derived cells - production of reactive antisera and absence of induction. J Neuroimmunol 1: 249–260. 7334081 10.1016/0165-5728(81)90029-1

[29] Gartlon J, Kinsner A, Bal-Price A, Coecke S, Clothier RH (2006) Evaluation of a proposed *in vitro* test strategy using neuronal and non-neuronal cell systems for detecting neurotoxicity. Toxicol In Vitro 20: 1569–1581. 16959468 10.1016/j.tiv.2006.07.009

[30] Schuliga M, Chouchane S, Snow ET (2002) Upregulation of glutathione-related genes and enzyme activities in cultured human cells by sublethal concentrations of inorganic arsenic. Toxicol Sci 70: 183–192. 12441363 10.1093/toxsci/70.2.183

[31] McConkey DJ (1998) Biochemical determinants of apoptosis and necrosis. Toxicol Lett 99: 157–168. 9862281 10.1016/s0378-4274(98)00155-6

[32] Marini AM, Nowak TS (2000) Metabolic effects of 1-methyl-4-phenylpyridinium (MPP+) in primary neuron cultures. Journal of Neuroscience Research 62: 814–820. 11107166 10.1002/1097-4547(20001215)62:6<814::AID-JNR8>3.0.CO;2-F

[33] Gabryel B, Adamek M, Pudelko A, Malecki A, Trzeciak HI (2002) Piracetam and vinpocetine exert cytoprotective activity and prevent apoptosis of astrocytes *in vitro* in hypoxia and reoxygenation. Neurotoxicol 23: 19–31. 10.1016/s0161-813x(02)00004-9 12164545

[34] Brecht S, Gelderblom M, Srinivasan A, Mielke K, Dityateva G (2001) Caspase-3 activation and DNA fragmentation in primary hippocampal neurons following glutamate excitotoxicity. Molecular Brain Research 94: 25–34. 11597762 10.1016/s0006-8993(01)02767-6

[35] Anderson ME (1985) Determination of glutathione and glutathione disulphide in biological samples. Methods in Enzymol 113: 548–555. 4088074 10.1016/s0076-6879(85)13073-9

[36] Coleman MD (2000) Use of *in vitro* methaemoglobin generation to study antioxidant status in the diabetic erythrocyte. Biochem Pharmac 60: 1409–1416. 10.1016/s0006-2952(00)00333-6 11020442

[37] Fritz R, Lanen C, Colas V, Leroux P (1997) Inhibition of methionine biosynthesis in *Botrytis cinerea* by the anilinopyrimidine fungicide pyrimethanil. Pest Sci 49: 40–46.

[38] Daniels A, Lucas JA (1995) Mode of action of the anilinopyrimidine fungicide pyrimethanil. 1. In-vivo activity against *Botrytis fabae* on broad bean (*Vicia faba*) leaves. Pest Sci 45, 33–41.

[39] Pose-Juan AE, Rial-Oteroa R, Paradelob M, López-Periagob JE (2011) Influence of the adjuvants in a commercial formulation of the fungicide “Switch” on the adsorption of their active ingredients: Cyprodinil and fludioxonil, on soils devoted to vineyard. J Hazard Mat 193: 288–295.. 10.1016/j.jhazmat.2011.07.074 21868160

[40] Pillonel CH, Meyer T (1997) Effect of phenylpyrroles on glycerol accumulation and protein kinase activity of *Neurospora crassa* . Pest Sci 49: 229–236.

[41] FAO/WHO (2005) Report of the joint meeting of the FAO panel of experts on pesticide residues in food and the environment and the WHO core assessment group on pesticide residues. Switzerland: Geneva. Available: http://whqlibdoc.who.int/publications/2006/9241665211_eng.pdf. Accessed 2012 July 13.

[42] United States Environmental Protection Agency: Pesticide Fact Sheet Cyprodinil: July 1998.

[43] Gradish AE, Scott-Dupree CD, Shipp L, Harris CR, Ferguson G (2010) Effect of reduced risk pesticides for use in greenhouse vegetable production on *Bombus impatiens* (Hymenoptera: Apidae). Pest Manag Sci 66: 142–146. 19757502 10.1002/ps.1846

[44] Ragan CI (1987) Structure of NAH-Ubiquinone reductase (complex-1). Curr Top Bioenerget 15: 1–161.

[45] Karadag H, Bilgin R (2010) Effect of Cyprodinil and Fludioxonil Pesticides on Human Superoxide Dismutase. Asian J Chem 22: 8147–8154.

[46] Wang W, Ghandi A, Liebes L, Louie SG, Hofman FM (2011) Effective conversion of irinotecan to SN-38 after intratumoral drug delivery to an intracranial murine glioma model *in vivo* Laboratory investigation. J Neurosurg 114: 689–694. 20415520 10.3171/2010.2.JNS09719

[47] Holden LJ, Coleman MD (2007) Assessment of the astrogliotic responses of three human astrocytoma cell lines to ethanol, trimethyltin chloride, and acrylamide. Toxicology 241: 75–83. 17875352 10.1016/j.tox.2007.08.083

[48] Holden LJ, Coleman MD (2008) Further preliminary assessment of three human glioma cell lines as models of human astrocytic toxicity *in vitro* . Env Toxicol Pharmacol 26: 290–296. 21791377 10.1016/j.etap.2008.05.009

[49] Reali C, Scintu F, Pillai R, Donato R, Michetti F (2005) S100B counteracts effects of the neurotoxicant trimethyltin on astrocytes and microglia. J Neurosci Res 81: 677–686. 15986416 10.1002/jnr.20584

[50] Figiel I, Fiedorowicz A (2002) Trimethyltin-evoked neuronal apoptosis and glia response in mixed cultures of rat hippocampal dentate gyrus: a new model for the study of the cell type-specific influence of neurotoxins. Neurotoxicology 23: 77–86. 12164551 10.1016/s0161-813x(02)00006-2

[51] Raicevica N, Mladenovica A, Perovica M, Harhajia L, Miljkovica D (2005) Iron protects astrocytes from 6-hydroxydopamine toxicity. Neuropharmacol 48: 720–731. 10.1016/j.neuropharm.2004.12.003 15814106

[52] Fernandez-Fernandez S, Almeida A, Bolanos JP (2012) Antioxidant and bioenergetic coupling between neurons and astrocytes. Biochem J 443: 3–11. 22417747 10.1042/BJ20111943

[53] Koprivica V, Regardie K, Wolff C, Fernall DR, Murphy JJ (2011) Aripiprazole protects cortical neurons from glutamate toxicity. Eur J Pharmacol 651: 73–76. 21093427 10.1016/j.ejphar.2010.10.064

[54] Zou DJ, Wang G, Liu JC, Dong MX, Li XM (2011) Beta-asarone attenuates beta-amyloid-induced apoptosis through the inhibition of the activation of apoptosis signal-regulating kinase 1 in SH-SY5Y cells. Pharmazie 66: 44–51. 21391434

[55] Jaworska-Feil L, Jantas D, Leskiewicz M, Budziszewska B, Kubera M (2010) Protective effects of TRH and its analogues against various cytotoxic agents in retinoic acid (RA)-differentiated human neuroblastoma SH-SY5Y cells. Neuropeptides 44: 495–508. 20869113 10.1016/j.npep.2010.08.004

[56] Yang W, Tiffany-Castiglione E, Lee MY, Son IH (2010) Paraquat induces cyclooxygenase-2 (COX-2) implicated toxicity in human neuroblastoma SH-SY5Y cells. Toxicol Lett 199: 239–246. 20851755 10.1016/j.toxlet.2010.09.005

[57] Coleman MD, Zilz TR, Griffiths HR, Woehrling EK (2008) A Comparison of the Apoptotic and Cytotoxic Effects of Hexanedione Derivatives on Human Non-Neuronal Lines and the Neuroblastoma Line SH-SY5Y. Basic & Clin Pharmacol Toxicol 102: 25–31. 10.1111/j.1742-7843.2007.00148.x 17973901

[58] Liu C, Wang S, Li L, Zhao H, Jiang S (2011) Analysis of cyprodinil in leek and pepper and its decline under field conditions. Environ Monit Assess 179: 209–215. 20963485 10.1007/s10661-010-1730-y

[59] Marian A, Oliva J, Garcia C, Navarro SN, Barba A (2003) Dissipation Rates of Cyprodinil and Fludioxonil in Lettuce and Table Grape in the Field and under Cold Storage Conditions. J. Agric. Food Chem. 51: 4708–4711. 14705900 10.1021/jf021222e

[60] Cabras P, Angioni A, Garau VL, Melis M, Pirisi FM (1997) Fate of Some New Fungicides (Cyprodinil, Fludioxonil, Pyrimethanil, and Tebuconazole) from Vine to Wine. J Agric Food Chem 45: 2708–2710.

[61] Lorenzin M (2007) Pesticide residues in Italian Ready-Meals and dietary intake estimation. J Environ Sci Health Part B 42: 823–833.. 10.1080/03601230701555021 17763040

[62] Ravelo-Pérez LM, Hernández-Borges J, Cifuentes A. Rodríguez-Delgado MA (2007) Multiple pesticide analysis in wine by MEKC combined with solid-phase microextraction and sample. Electrophoresis 28: 4072–4081.. 17957661 10.1002/elps.200700251

[63] Ravelo-Pérez LM, Hernández-Borges J, Cifuentes A (2007) Rodríguez-Delgado MA. MEKC combined with SPE and sample stacking for multiple analysis of pesticides in water samples at the ng/L level. Electrophoresis 28: 1805–1814.. 17476718 10.1002/elps.200600526

